# Prospective Longitudinal Characterization of the Relationship between Diabetes and Cardiac Structural and Functional Changes

**DOI:** 10.1155/2022/6401180

**Published:** 2022-02-08

**Authors:** Amrit Chowdhary, Nicholas Jex, Sharmaine Thirunavukarasu, Amanda MacCannell, Natalie Haywood, Altaf Almutairi, Lavanya Athithan, Manali Jain, Thomas Craven, Arka Das, Noor Sharrack, Christopher E. D. Saunderson, Anshuman Sengupta, Lee Roberts, Peter Swoboda, Richard Cubbon, Klaus Witte, John Greenwood, Sven Plein, Eylem Levelt

**Affiliations:** ^1^University of Leeds, Multidisciplinary Cardiovascular Research Centre and Biomedical Imaging Science Department, Leeds Institute of Cardiovascular and Metabolic Medicine, Leeds LS29JT, UK; ^2^University of Leeds, Discovery and Translational Science Department, Leeds Institute of Cardiovascular and Metabolic Medicine, Leeds LS29JT, UK; ^3^National Institute for Health Research Biomedical Research Centre—Department of Cardiovascular Sciences, University of Leicester, Glenfield Hospital, Groby Road, Leicester LE3 9QP, UK; ^4^Department of Cardiology, Leeds Teaching Hospitals NHS Trust, Great George Street, Leeds LS13EX, UK

## Abstract

**Objectives:**

In a cohort of type 2 diabetic (T2D) patients who underwent baseline cardiac magnetic resonance (CMR) and biomarker testing, during a median follow-up of 6 years, we aimed to determine longitudinal changes in the phenotypic expression of heart disease in diabetes, report clinical outcomes, and compare baseline clinical characteristics and CMR findings of patients who experienced major adverse cardiovascular events (MACE) to those remaining MACE free.

**Background:**

T2D increases the risk of heart failure (HF) and cardiovascular mortality. The long-term impact of T2D on cardiac phenotype in the absence of cardiovascular disease and other clinical events is unknown.

**Methods:**

Patients with T2D (*n* = 100) with no history of cardiovascular disease or hypertension were recruited at baseline. Biventricular volumes, function, and myocardial extracellular volume fraction (ECV) were assessed by CMR, and blood biomarkers were taken. Follow-up CMR was repeated in those without interim clinical events after 6 years.

**Results:**

Follow-up was successful in 83 participants. Of those, 29 experienced cardiovascular/clinical events (36%). Of the remaining 59, 32 patients who experienced no events received follow-up CMR. In this cohort, despite no significant changes in blood pressure, weight, or glycated hemoglobin, significant reductions in biventricular end-diastolic volumes and ejection fractions occurred over time. The mean ECV was unchanged. Baseline plasma high-sensitivity cardiac troponin T (hs-cTnT) was significantly associated with a change in left ventricular (LV) ejection fraction. Patients who experienced MACE had higher LV mass and greater LV concentricity than those who remained event free.

**Conclusions:**

T2D results in reductions in biventricular size and systolic function over time even in the absence of cardiovascular/clinical events.

## 1. Introduction

Cardiovascular disease represents the primary cause of death in type 2 diabetic patients (T2D) [1]. Although T2D is recognized as a strong risk factor for atherosclerosis-related events, heart failure (HF) is the commonest initial presentation of cardiovascular disease in T2D [1–3]. The risk of developing HF is increased 2.4-fold in men and 5-fold in women with T2D compared with age-matched controls [4], and the combination of T2D and HF is associated with a 4- to 6-fold higher mortality [5, 6]. The early detection of adverse subclinical myocardial structural and functional alterations associated with progressive myocardial dysfunction might offer the opportunity of early initiation of disease-modifying pharmacological therapies prior to the onset of overt HF [7].

Cardiac magnetic resonance imaging (CMR) is the reference standard for the assessment of cardiac volumes, mass, and function [8]. Using CMR, patients with T2D have been extensively phenotyped with a nuanced description of disease burden [9, 10]. However, to our knowledge no CMR study to date has examined longitudinal changes in biventricular structure and function in patients with T2D with no prior cardiovascular disease.

In this longitudinal observational study, we tested the hypothesis that T2D would be associated with a progressive decline in biventricular systolic function even in a cohort of diabetic patients with no prior cardiovascular disease or interim major adverse cardiovascular events (MACE) during the follow-up period. We also sought to report clinical outcomes and compare demographic, clinical, and biochemical variables, and CMR and plasma biomarkers measured at baseline between those patients who experienced MACE and those who remained free of MACE during the follow-up period.

## 2. Methods

Using CMR at two time points baseline visit and end of the study, we performed a prospective longitudinal study in a cohort of ethnically diverse, asymptomatic patients with T2D with no history or evidence on examination of cardiovascular disease. Participants who remained asymptomatic and free of MACE or any other new clinical comorbidity were invited for a second CMR scan after 6 years.

### 2.1. Participants

Recruitment was performed from primary care health centers in Leeds, the United Kingdom. One hundred participants with T2D were recruited at baseline [11]. The results of this initial study have been previously published [11, 12]. For this study, all surviving T2D participants who could be contacted and remained eligible were offered a follow-up research visit for a repeat CMR scan.

### 2.2. Inclusion and Exclusion Criteria

Asymptomatic adult patients with a diagnosis of T2D (diagnosed according to the World Health Organization criteria) [13] with the ability to provide informed written consent were recruited at baseline. Patients were excluded if they had a previous diagnosis of cardiovascular disease (previous cardiac surgery, angioplasty, myocardial infarction, angina, moderate or above valvular heart disease, and atrial fibrillation(AF)), hypertension (resting systolic blood pressure (BP) >140 mmHg and diastolic BP >90 mmHg on 24-hour ambulatory BP monitoring), contraindications to CMR, ischemic changes in 12-lead electrocardiogram (ECG), renal impairment (estimated glomerular filtration rate (eGFR) below 30 mL/min/1.73 m^2^), or if they were using insulin. After 6 years, surviving participants who remained asymptomatic, with no MACE, other diabetic complications, or important comorbidity (such as inflammatory disease or malignancy) were invited for a second CMR study.

### 2.3. Baseline Clinical Assessment

At baseline and at the follow-up visit, height and weight were recorded and body mass index (BMI) was calculated. A fasting blood sample was taken from each participant at baseline for assessments of full blood count (FBC), eGFR, fasting glucose, glycated hemoglobin (HbA1c), high-sensitivity cardiac troponin T (hs-cTnT), and N-terminal prohormone B-type natriuretic peptide (NT-proBNP) levels. All participants underwent resting ECG, and all had 24-hour BP monitoring at baseline to exclude undiagnosed hypertension [11]. At the follow-up study visit, brachial BP was recorded as an average of 3 supine measures taken over 10 minutes (Dinamap 1846 SX, Critikon Corp), a fasting blood sample was obtained for repeated assessments of FBC, eGFR, glucose, HbA1c, and lipids, and a resting ECG was recorded.

### 2.4. Cardiac Magnetic Resonance Imaging

Imaging was performed on a 3.0 Tesla magnetic resonance system both at baseline and at year 6 CMR study visit. The baseline CMR protocol has been previously described [11]. Follow-up scans were performed using a matching imaging protocol.

**Figure 1:**
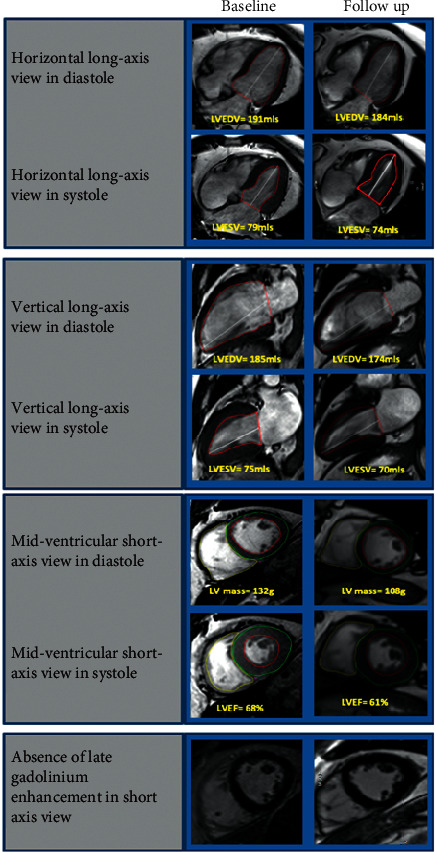
Representative examples of CMR imaging. Representative examples of cine images (horizontal long axis, vertical long axis, and mid-ventricular short axis in diastole and systole) and late gadolinium enhancement imaging (mid-ventricular short axis) in patients with T2D at baseline and follow-up.

Images for biventricular and left atrial (LA) volumes and function were acquired using a steady-state free precession (SSFP) sequence with breath holding at end expiration in multiple orientations (Figure 1). Adenosine stress myocardial perfusion CMR was performed to rule out significant epicardial coronary artery stenosis [14]. Pharmacological stress was achieved with adenosine infusion at 140 mcg/kg/min for a minimum of 3 mins, and an intravenous bolus of 0.075 mmol/kg gadobutrol (Gadovist®, Bayer Pharma, Berlin, Germany) was administered for each stress and rest perfusion imaging sequence. Visual analysis of the perfusion images was performed by one reporter (EL, with >8 years of CMR experience and level 3 CMR accreditation). Ischemia was defined as a territory with a perfusion defect during stress [14]. Late gadolinium enhancement (LGE) imaging was performed in matching LV short-axis planes >8 minutes after contrast administration to exclude the presence of previous silent myocardial infarction or regional fibrosis.

All image analyses were performed off-line by AC (with 2 years of CMR experience) in a blinded fashion, and all scan contours were subsequently reviewed by EL using cvi42 software (Circle Cardiovascular Imaging, Calgary, Canada). Baseline and follow-up images were analyzed in a random order after the second visit by investigators blinded to any other data. Biventricular volumes and ejection fraction (EF) were obtained from contouring the endocardial and epicardial borders in diastole and systole on the SSFP short-axis stacks. The LA volume and EF were calculated using the biplane area-length method in the horizontal and vertical long axes as previously described [8]. Using cvi42 Tissue Tracking software, global longitudinal strain (GLS), and a marker of diastolic function, LV diastolic strain measurements were performed from balanced SSFP short-axis and 2-chamber and 4-chamber long-axis cine images, to calculate circumferential peak early diastolic strain rate (PEDSR) and longitudinal PEDSR [10].

### 2.5. Statistical Analysis

Statistical analysis was performed using SPSS (IBM SPSS statistics, version 26.0). Categorical data were compared with Pearson's chi-square test. Continuous variables were checked for normality using the Shapiro-Wilk test and are presented as mean ± SD. Comparisons of CMR data between baseline and follow-up were performed with a two-tailed paired *t*-test. Bivariate correlations were performed using Pearson's correlation coefficient. The relationships of change in left ventricular ejection fraction (LVEF) and right ventricular ejection fraction (RVEF) (ΔLVEF and ΔRVEF, respectively) with age, BMI, HBA_1_c, fasting glucose, resting BP and heart rate (HR), NT-proBNP, and hs-cTnT were analyzed using multiple logistic regression. A *p*-value of ≤0.05 was considered statistically significant.

### 2.6. Ethical Considerations

The study was approved by the National Research Ethics Committee (Ref: 13/YH/0098), and informed written consent was obtained from each participant. The follow-up assessment was given additional ethical approval (Ref: 18/YH/0168). Participants were asked to sign a second consent form for the follow-up scan.

## 3. Results

### 3.1. Participant Characteristics and Clinical Outcomes

**Figure 2:**
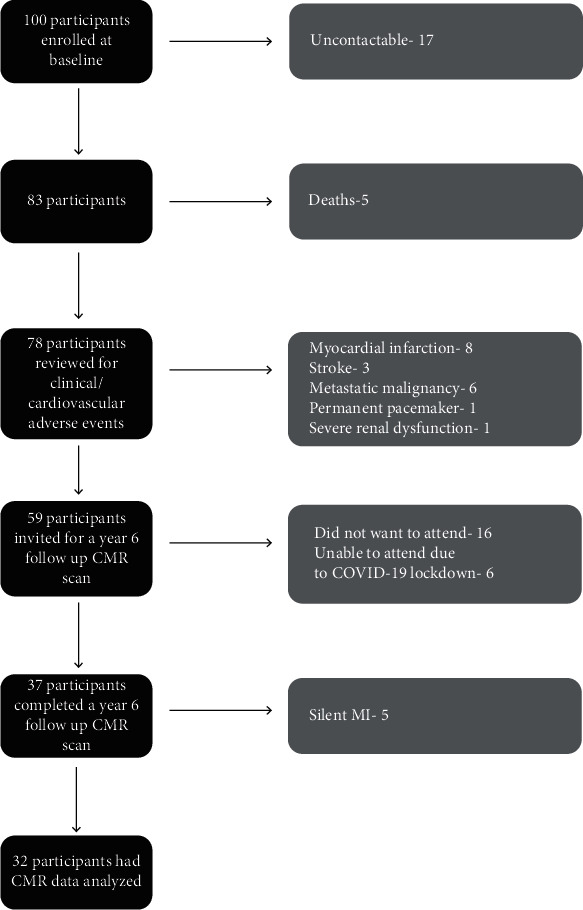
Recruitment flowchart. Recruitment flowchart demonstrating recruitment and follow-up pathway for the participants (CMR-cardiac magnetic resonance; COVID-19-coronavirus disease; MI-myocardial infarction).

**Figure 3:**
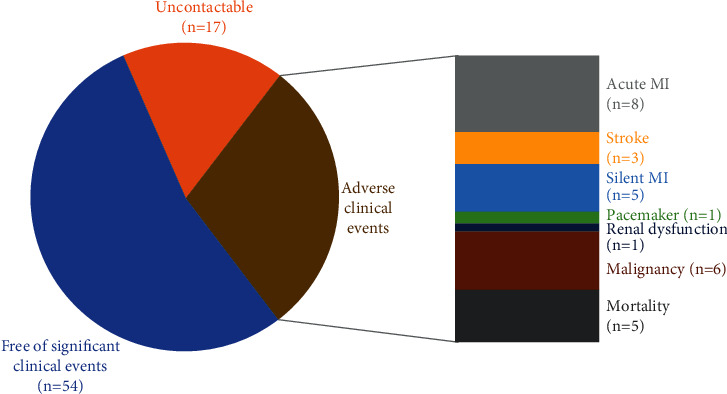
Major adverse cardiovascular event rates. The major adverse cardiovascular event rate (MI, angina, revascularization, CVA, and death) during the 6-year follow-up period, including the patients with a silent MI, amounted to 25% in this study with an overall clinical event rate of 35%.

Clinical outcomes of the baseline cohort were determined after a median follow-up of 6.3 years (interquartile range (IQR): 6.05–6.53 years) using electronic health records systems, and symptom status was determined by phone assessments (Figure 1). Demographics, clinical, and biochemical data are shown in Table 1. Of the hundred participants with T2D recruited at baseline (82 men, mean age 61 ± 11 years, median diabetic duration 4.1 years (IQR: 1.4–7 years), 17 participants were uncontactable (Figure 2). The healthcare records of the remaining 83 participants revealed that 5 participants (6%) had died during the follow-up period (one due to acute coronary syndrome (ACS)), 8 participants (9.6%) had survived an ACS, 3 participants (3.8%) had a cerebrovascular accident, 6 participants (7.7%) developed a malignancy, 1 participant (1.3%) had a permanent pacemaker implanted for a high-grade atrioventricular block, and 1 participant (1.3%) developed significant renal dysfunction (Figure 2). These participants were not invited back for a follow-up CMR scan. Of the remaining 59 participants (76%) with T2D who remained free of MACE invited for a repeat CMR scan, 16 participants declined, and a further 6 participants were unable to attend for their research visit due to the coronavirus pandemic (Figure 2). Hence, 37 participants completed a second CMR scan. Of these, 5 were found to have suffered a silent MI as evidenced by subendocardial hyperenhancement on LGE and were excluded from further analysis leaving a study population of 32 participants. About 25% of the original study population had suffered a major adverse cardiovascular event (MI, angina, revascularization, stroke, and cardiovascular mortality) during the 6-year follow-up period (Figure 3) with an overall clinical event rate of 35%.

### 3.2. Baseline to Follow-Up Demographics and Medical Therapy

In the 32 patients comprising this study cohort, 29 were men, the mean age was 64 ± 9 years, and median diabetic duration was 11.9 years (IQR: 11.8–12.3 years). There were no significant differences in resting HR and BP, glycemic control, or BMI between the baseline and follow-up measurements (Table 2). Glucose-lowering treatment had been altered for the majority of patients between baseline and follow-up. While the proportion of patients on a biguanide reduced from 88% to 69% (*p*=0.01), the proportion taking a sodium-glucose cotransporter-2 (SGLT2) inhibitor increased from none to 12% (*p*=0.03). The number of participants on sulfonylureas, thiazolidinediones, gliptins, aspirin, or statins did not significantly change during the follow-up period.

None of the patients were on an angiotensin-converting enzyme inhibitor (ACEI) or an angiotensin receptor blocker (ARB) therapy at baseline as per recruitment criteria of the initial study [11], whereas 13 participants (41%) were receiving this therapy at the time of follow-up visit (Table 2).

### 3.3. Cardiac Geometry, Function, and Myocardial Scarring

**Figure 4:**
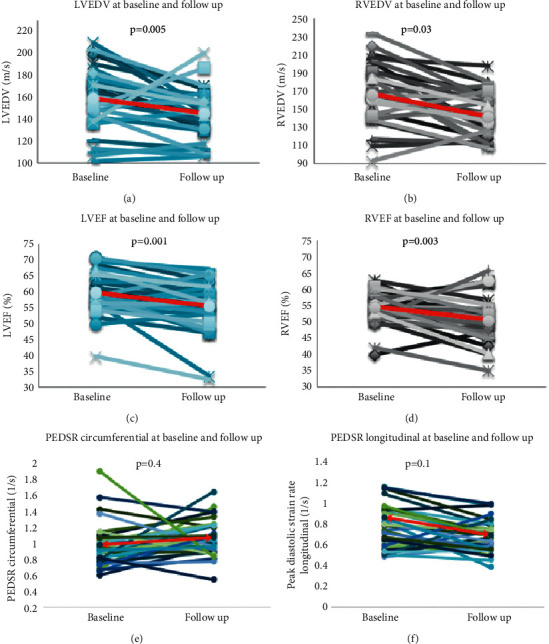
Comparison of imaging parameters at baseline and follow-up. Comparison between the left ventricular end-diastolic volume (LVEDV), right ventricular end-diastolic volume (RVEDV), left ventricular ejection fraction (LVEF), right ventricular ejection fraction (RVEF), peak diastolic strain rate (PEDSR) circumferential and PEDSR longitudinal at baseline, and year 6 follow-up scans (line in red indicates mean values for each variable).

The CMR results of the 32 participants at baseline and follow-up are shown in Table 3. At follow-up, there was a reduction in cardiac size with reduced biventricular end-diastolic volumes (Figures 4(a) and 4(b)) and a deterioration of biventricular systolic function (mean LVEF 60 ± 7% vs. 55 ± 8%, *p*=0.0001; mean RVEF 55 ± 5% vs. 51 ± 7%, *p*=0.003) (Figures 4(c) and 4(d)) with reductions in stroke volumes. In keeping with the reduction in cardiac size, the LV mass and mass index were reduced at follow-up compared with baseline (*p*=0.01 and *p*=0.04, respectively). There was no change in LV PEDSR over time (Figures 4(e) and 4(f)). Consistent with the changes in LVEF, GLS was numerically reduced from baseline over time; however, this trend did not reach statistical significance. In keeping with the reductions in biventricular volumes, LA volumes were also decreased, but there was no change in LA function over time.

Mean change in LVEF was the same in those taking and those not taking renin-angiotensin-aldosterone blocking agents.

The presence of new mid-wall fibrosis in a nonischemic pattern was detected in only one patient at follow-up. There were no changes in mean ECV between baseline and follow-up (*p*=0.3).

### 3.4. Comparison of CMR Features, Plasma Biomarkers, and Biochemistry at Baseline between Patients Who Experienced Cardiovascular Events and Those Who Remained Asymptomatic

There were no differences in baseline hs-cTnT and NT-proBNP biomarker levels or clinical and biochemical variables at baseline in participants who experienced MACE (angina, myocardial infarction, revascularization, cerebrovascular accident, and cardiovascular mortality) compared to those who did not (Table 4). However, patients who experienced MACE during the follow-up period had higher LV mass, LV mass indexed to body surface area, and a higher LV mass-to-LV EDV ratio indicating a greater concentric remodeling of the LV at baseline compared to those remaining asymptomatic and event free during the follow-up (Table 5).

### 3.5. Associations of the Change in Myocardial Function and Baseline Variables

There were no associations between changes in cardiac function and the baseline clinical variables. Although there were also no associations between laboratory variables of glucose management or NT-proBNP, there was a significant correlation between the change in LVEF and baseline plasma hs-cTnT (*R* = −0.44, *p*=0.01). There was no such association for change in RVEF.

## 4. Discussion

Despite the epidemiologically established link between T2D and congestive cardiac failure [2], longitudinal cardiac structural and functional changes in asymptomatic patients with T2D who remain free of cardiovascular events have not been explored before. In a cohort of ethnically diverse, asymptomatic patients with T2D with no history of prior cardiovascular disease, this study has shown for the first time that T2D is associated with clinically relevant adverse changes in biventricular function at follow-up after 6 years even in the absence of cardiovascular events, cardiac ischemia, or other predisposing factors such as hypertension. The present data have also shown that baseline glucose control seems to have no effect, although plasma hs-cTnT does predict the magnitude of the subsequent change in LVEF. Finally, underscoring the prognostic relevance of changes in LV mass and LV geometry in diabetes, this study has also shown higher LV mass and greater LV concentric remodeling at baseline in patients who experienced MACE during the follow-up compared to those who remained asymptomatic and event free. There were no other significant differences in clinical or biochemical variables between the two groups, suggesting that the adverse cardiovascular events in T2D are not limited to patients with poor glycemic, BP, or weight control.

### 4.1. Longitudinal Morphological Alterations in Type 2 Diabetes

Our results show that cardiac size and mass decrease over time in patients with T2D, while biventricular function deteriorates. In contrast to our findings in patients with T2D, in healthy aging LVEF remains static or increases over time as shown by multiple studies [15–19].

In this study, in 30% of the patients on the year 6 CMR scan, LVEF levels dropped below the normal range (<50%) despite the asymptomatic status of these patients [20]. Supporting our findings, multiple studies showed that even in asymptomatic individuals with T2D, there is a high prevalence of LV systolic and diastolic dysfunction [10, 21]. The American Heart Association has classified asymptomatic individuals with impaired cardiac function as having stage B HF [22]. These patients remain at risk for significant cardiovascular morbidity and mortality and experience a 5-fold increase in the risk of subsequent symptomatic HF development [23]. As stage B HF is a precursor to clinical HF, earlier identification of the cardiovascular manifestations of stage B HF may permit earlier diagnosis and treatment of patients at higher risk.

### 4.2. Relationship of Glycemic Control, Blood Pressure Control, Body Weight Changes, and Longitudinal Cardiac Functional Changes in Type 2 Diabetes

There were no changes in mean HbA1c, systolic and diastolic BP, resting HR, or BMI at follow-up. Moreover, we detected no relationship between the baseline systolic or diastolic BP, HR, BMI, HBA1c, and glucose levels with the change in LVEF and RVEF over time (ΔLVEF and ΔRVEF, respectively). This lack of association between glycemic control and cardiac functional decline supports the notion that there are more central mechanisms to HF pathophysiology in diabetes than glycemic control. While a few studies demonstrated a positive impact of metabolic control on ventricular function [24, 25], most previous studies failed to demonstrate any favorable changes in cardiac function despite improvements in glycemic control [26, 27]. Interestingly, we have not detected any significant changes in the diastolic function in this cohort despite the aging process. This is likely to be a consequence of the normotensive status of the cohort at baseline with no significant changes in systolic or diastolic BP assessments over time despite the aging process.

### 4.3. Relationship of Plasma Biomarkers (High-Sensitivity Cardiac Troponin T and N Terminal Pro B-type Natriuretic Peptide) and Longitudinal Cardiac Functional Changes in Type 2 Diabetes

High-sensitivity cardiac troponin isoforms are unique to the cardiac myocyte and are objective, quantifiable, and sensitive biomarkers for detecting cardiac injury [28]. They are predictors of cardiovascular morbidity and mortality risk in population-based studies besides their role as the cornerstone for the diagnosis of acute myocardial infarction [29]. We show in this study for the first time that there is a significant association between the plasma hs-cTnT measured at baseline with change in LVEF over time, highlighting a potentially important role for hs-cTnT as a biomarker for assessing HF risk in patients with T2D. A recent study has shown that lifestyle factors, such as smoking, diet, and physical activity, are associated with changes in high-sensitivity cardiac troponin levels, suggesting that lifestyle modifications may be able to affect changes in troponin and be beneficial in reducing mortality risk. As an easily obtainable plasma biomarker, hs-cTnT may be of great assistance in the incremental risk stratification of patients with T2D into high-risk and low-risk subgroups [28].

Our study does not suggest a similar role for NT-proBNP in asymptomatic patients with T2D with no known cardiovascular disease. A previous diabetic study did, however, demonstrate an independent correlation of NT-proBNP with the short-term prognosis of cardiovascular events [30]. The discrepancy between the two studies might have resulted from the distinct populations investigated. While Huelsmann et al. [30] have not excluded symptomatic patients, patients with ischemic heart disease, AF, or other significant cardiovascular diseases, in order to better characterize the occult heart disease in diabetes we have excluded these comorbidities and symptomatic patients.

### 4.4. Left Ventricular Geometry and Major Adverse Cardiovascular Events in Type 2 Diabetes

LV mass is a strong and independent predictor of subsequent cardiovascular events, including myocardial infarction, HF, and mortality [31]. While the precise underlying mechanism of LV hypertrophy and concentric LV remodeling in the absence of significant hypertension remains unclear, it has been suggested that T2D induces LV mass enlargement through metabolic, and not hemodynamic pathways [32]. Supporting this, a recent study has shown that treatment with selective SGLT2 inhibitor empagliflozin was associated with significant reductions in LV mass, which may account in part for the beneficial cardiovascular outcomes of empagliflozin [33].

## 5. Study Limitations

The present data are in a modest number of patients, recruited to a single study site. The study was not powered to assess the potential association of the treatments and the CMR findings with regression analysis. However, the longitudinal nature of the study allowed paired analysis of images that, to minimize bias, were randomized in time and by subject. Moreover, the image data of a random sample of subjects were evaluated by two investigators to demonstrate good inter- and intraobserver reproducibility.

Another limitation is the small number of female participants as only a smaller proportion agreed to return for a second scan. While diabetes has been consistently found to be a stronger risk factor for heart disease in women compared to men [2], in this study we show that biventricular reductions in systolic function occur over time even in a predominantly male population.

## 6. Conclusions

Even in the absence of overt clinical CAD, significant valvular disease, uncontrolled hypertension, or change in BMI, T2D resulted in a significant reduction in cardiac size and biventricular systolic function over time. Plasma hs-cTnT measured at baseline was associated with the magnitude of change in LV systolic function suggesting that hs-cTnT could play a role in identifying patients with T2D at higher risk for heart failure. Patients who experienced MACE during the follow-up exhibited higher LV mass and greater LV concentric remodeling at baseline compared to those who remained asymptomatic and event free.

## Figures and Tables

**Table 1:** Clinical and biochemical characteristics.

Variable	Baseline total participants (*n* = 100)	Follow-up (*n* = 32)	*P* value
Age, years	61 ± 11	64 ± 11	0.2
BMI, kg/m^2^	29 ± 4	27 ± 4	0.02^*∗*^
Male, *n* (%)	82 (82)	29 (91)	0.2
Diabetes duration, years	5.0 ± 4.4	10.9 ± 1.3	0.0001^*∗*^
Smoking, *n* (%)	6 (6)	2 (6)	0.9
Heart rate, bpm	71 ± 12	68 ± 12	0.2
Systolic blood pressure, mmHg	131 ± 15	129 ± 16	0.5
Diastolic blood pressure, mmHg	73 ± 9	74 ± 7	0.6
Plasma fasting glucose, mmol/L	9.9 ± 4.1	9.4 ± 3.8	0.5
Glycated hemoglobin, mmol/mol	63 ± 20	64 ± 18	0.7
Total cholesterol, mmol/L	4.4 ± 1.1	4.5 ± 1.2	0.7
HDL, mmol/L	1.19 ± 0.35	1.36 ± 0.38	0.03^*∗*^
LDL, mmol/L	2.60 ± 0.98	2.59 ± 1.21	0.9
Medications, *n* (%)			
Metformin	87 (87)	22 (69)	0.01^*∗*^
Sulfonylurea	33 (33)	13 (40)	0.4
Aspirin	18 (18)	9 (28)	0.2
Statin	69 (69)	23 (72)	0.7
ACEI	0 (0)	13 (40)	0.001^*∗*^
ARB	0 (0)	3 (9)	0.0001^*∗*^

Values are mean ± standard deviations or percentages. ^*∗*^signifies *P* ≤ 0.05. *n*-numbers; BMI-body mass index; kg-kilogram; m-meter; bpm-beats per minute; mmHg-millimeters of mercury; mmol-millimoles; L-liters; mol-moles; HDL-high-density lipoprotein; LDL-low-density lipoprotein; ACEI-angiotensin-converting enzyme inhibitor; ARB-angiotensin receptor blocker.

**Table 2:** Clinical and biochemical characteristics of the participants who had baseline and year 6 follow-up CMR scans.

Variable	Baseline (*n* = 32)	Follow-up (*n* = 32)	*P* value
Age, years	58 ± 11	64 ± 11	0.03^*∗*^
BMI, kg/m^2^	28 ± 4	27 ± 4	0.1
Male, *n* (%)	29 (91)	29 (91)	1.0
Smoker, *n* (%)	1 (3)	2(6)	0.6
Diabetes duration, years	5.1 ± 1.2	10.9 ± 1.3	0.0001^*∗*^
Heart rate, bpm	72 ± 13	68 ± 12	0.3
Systolic blood pressure, mmHg	131 ± 16	129 ± 16	0.7
Diastolic blood pressure, mmHg	73 ± 10	74 ± 7	0.4
Plasma fasting glucose, mmol/L	8.5 ± 3.5	9.4 ± 3.8	0.3
Glycated hemoglobin, mmol/mol	61 ± 15	64 ± 18	0.13
hs-cTnT, ng/ml	7.35 ± 5.14	—	
Total cholesterol, mmol/L	4.5 ± 1.3	4.5 ± 1.2	1.0
HDL, mmol/L	1.24 ± 0.31	1.36 ± 0.38	0.2
LDL, mmol/L	2.86 ± 1.19	2.59 ± 1.21	0.4
Medications *n* (%)			
Metformin, *n* (%)	28 (88)	22 (69)	0.01^*∗*^
Sulfonylurea, *n* (%)	12 (38)	13 (40)	0.3
Gliptin, *n* (%)	3 (9)	7 (22)	0.1
Thiazolidinediones, *n* (%)	0	2 (6)	0.1
SGLT2 inhibitors, *n* (%)	0	4 (12)	0.03^*∗*^
Aspirin, *n* (%)	7 (22)	9 (28)	0.1
Statin, *n* (%)	22 (69)	23 (72)	0.3
ACEI, *n* (%)	0 (0)	13 (40)	0.0001^*∗*^
ARB, *n* (%)	0 (0)	3 (9)	0.08

Values are mean ± standard deviations or percentages. ^*∗*^signifies *P* ≤ 0.05. CMR-cardiovascular magnetic resonance imaging; n-numbers; BMI-body mass index; kg-kilogram; m-meter; bpm-beats per minute; mmHg-millimeters of mercury; mmol-millimoles; L-liters; mol-moles; hs-cTnT-high-sensitivity cardiac troponin-T; ng-nanograms; HDL-high-density lipoprotein; LDL-low-density lipoprotein; SGLT2-sodium glucose cotransporter 2; ACEI-angiotensin-converting enzyme inhibitor; ARB-angiotensin receptor blocker.

**Table 3:** CMR findings.

Variable	Baseline (*n* = 32)	Follow-up (*n* = 32)	*P* value
LV end-diastolic volume (ml)	159 ± 29	145 ± 22	0.005^*∗*^
LV end-diastolic volume index (ml/m^2^)	78 ± 12	73 ± 10	0.02^*∗*^
LV end-systolic volume (ml)	64 ± 16	65 ± 19	0.5
LV end-systolic volume index (ml/m^2^)	31 ± 7	33 ± 9	0.3
LV stroke volume (ml)	95 ± 20	80 ± 14	0.001^*∗*^
LV ejection fraction (%)	60 ± 7	55 ± 8	0.0001^*∗*^
ΔLVEF (%)	−(5.66 ± 4.38)		
LV mass (gm)	102 ± 17	94 ± 16	0.01^*∗*^
LV mass index (gm/m^2^)	51 ± 8	47 ± 8	0.04^*∗*^
LV mass to LV end-diastolic volume (gm/ml)	0.65 ± 0.12	0.66 ± 0.14	0.8
Global longitudinal strain (%, negative)	13.06 ± 2.05	11.74 ± 2.54	0.8
Peak diastolic circumferential strain rate (1/s)	0.98 ± 0.28	1.04 ± 0.23	0.4
Peak diastolic longitudinal strain rate (1/s)	0.86 ± 0.19	0.69 ± 0.17	0.1
RV end-diastolic volume (ml)	166 ± 33	142 ± 25	0.03^*∗*^
RV end-diastolic volume index (ml/m^2^)	82 ± 14	71 ± 12	0.0001^*∗*^
RV end-systolic volume (ml)	76 ± 18	70 ± 16	0.05^*∗*^
RV end-systolic volume index (ml/m^2^)	37 ± 8	35 ± 8	0.1
RV stroke volume (ml)	91 ± 20	72 ± 15	<0.0001^*∗*^
RV ejection fraction (%)	55 ± 5	51 ± 7	0.003^*∗*^
ΔRVEF (%)	−(6.69 ± 4.15)		
LA maximum volume (ml)	88 ± 17	67 ± 21	0.0001^*∗*^
LA ejection fraction (%)	58 ± 6	56 ± 9	0.4
Extracellular volume (%)	24.96 ± 3.02	24.10 ± 2.66	0.3

Values are mean±standard deviations or percentages. ^*∗*^signifies *P* ≤ 0.05. CMR-cardiac magnetic resonance imaging; n-numbers; LV-left ventricle; ml-milliliters; m-meter; ΔLVEF-change in LV ejection fraction; gm-grams; s-seconds; RV-right ventricle; ΔRVEF-change in RV ejection fraction; LA-left atrium.

**Table 4:** Clinical and biochemical characteristics at baseline of the participants with and without MACE (angina, myocardial infarction, revascularization, and cerebrovascular accident) at follow-up.

Variable	No MACE (*n* = 65)	MACE (*n* = 18)	*P* value
Age, years	66 ± 11	65 ± 9	0.7
BMI (baseline), kg/m^2^	29 ± 4	28 ± 3	0.3
Male, %	55 (85)	17 (94)	0.2
Diabetes duration, years	9.9 ± 4.6	10.6 ± 3.8	0.6
Smoker, *n* (%)	6 (10)	6 (33)	0.01^*∗*^
Systolic blood pressure, mmHg	131 ± 15	132 ± 14	0.8
Diastolic blood pressure, mmHg	72 ± 9	74 ± 9	0.4
Glycated hemoglobin, mmol/mol	62 ± 21	71 ± 20	0.1
Troponin T, ng/L	7.6 ± 5.8	7.0 ± 3.6	0.7
NT-proBNP, pg/ml	72 ± 129	39 ± 42	0.3
Total cholesterol, mmol/L	4.3 ± 1.1	4.6 ± 1.3	0.3
LDL, mmol/L	2.6 ± 0.9	2.7 ± 1.6	0.7
Medications, *n* (%)			
Metformin, *n* (%)	57 (88)	15 (83)	0.6
Sulfonylurea, *n* (%)	21 (32)	6 (33)	0.9
Gliptins, *n* (%)	7 (11)	4 (22)	0.2
Thiazolidinediones, *n* (%)	0 (0)	0 (0)	—
SGLT2 inhibitors, *n* (%)	0 (0)	0 (0)	—
Aspirin, *n* (%)	15 (23)	3 (17)	0.6
Statin, *n* (%)	47 (72)	13 (72)	0.9
ACE-I, *n* (%)	0 (0)	0 (0)	—
ARB, *n* (%)	0 (0)	0 (0)	—
Beta-blockers, *n* (%)	3 (4)	1 (5)	0.9
Calcium channel blockers, *n* (%)	6 (8)	3 (17)	0.4

Values are mean ± standard deviations or percentages. ^*∗*^signifies *P* ≤ 0.05. MACE-major adverse cardiovascular events; n-numbers; BMI-body mass index; kg- kilograms; m-meters; mmHg-millimeters of mercury; mmol-millimoles, mol-moles; ng-nanograms; L-liters; NT-pro BNP-N-terminal prohormone B-type natriuretic peptide; pg-picograms; ml-milliliters; LDL-low-density lipoprotein; SGLT2-sodium-glucose cotransporter 2; ACE-I-angiotensin-converting enzyme inhibitor; ARB-angiotensin receptor blocker.

**Table 5:** CMR findings at baseline of the participants with and without MACE (angina, myocardial infarction, revascularization, and cerebrovascular accident) at follow-up.

	No MACE (*n* = 65)	MACE (*n* = 18)	*P* value
LV end-diastolic volume (ml)	146 ± 35	147 ± 35	0.9
LV end-diastolic volume index (ml/m^2^)	73 ± 15	72 ± 14	0.8
LV end-systolic volume (ml)	59 ± 21	58 ± 22	0.9
LV end-systolic volume index (ml/m^2^)	29 ± 9	28 ± 9	0.9
LV stroke volume (ml)	87 ± 20	89 ± 16	0.7
LV ejection fraction (%)	60 ± 7	61 ± 6	0.6
LV mass (gm)	102 ± 23	116 ± 25	0.02^*∗*^
LV mass index (gm/m^2^)	51 ± 9	57 ± 10	0.01^*∗*^
LV mass to LV end-diastolic volume (gm/ml)	0.72 ± 0.13	0.79 ± 0.15	0.05^*∗*^
RV end-diastolic volume (ml)	151 ± 37	147 ± 31	0.6
RV end-diastolic volume index (ml/m^2^)	76 ± 17	72 ± 14	0.3
RV end-systolic volume (ml)	68 ± 21	67 ± 17	0.8
RV end-systolic volume index (ml/m^2^)	34 ± 10	33 ± 7	0.7
RV stroke volume (ml)	83 ± 19	80 ± 19	0.5
RV ejection fraction (%)	55 ± 5	54 ± 7	0.5

Values are mean± standard deviations or percentages. ^*∗*^signifies *P* ≤ 0.05. CMR-cardiovascular magnetic resonance; MACE-major adverse cardiovascular events; LV-left ventricle; ml-milliliters, m-meter; gm-grams; RV-right ventricle.

## Data Availability

Data can be made available on request through e-mail to the corresponding author.

## Abbreviations List

ACEI: Angiotensin-converting enzyme inhibitor
ARB: Angiotensin receptor blocker
AF: Atrial fibrillation
BMI: Body mass index
BP: Blood pressure
CAD: Coronary artery disease
CMR: Cardiovascular magnetic resonance imaging
ECG: Electrocardiogram
ECV: Extracellular volume fraction
EF: Ejection fraction
eGFR: Estimated glomerular filtration rate
HBA1c: Glycated hemoglobin
HDL: High-density lipoprotein
HF: Heart failure
hs-cTnT: High-sensitivity cardiac troponin T
IQR: Interquartile range
LA: Left atrial
LGE: Late gadolinium enhancement
LV: Left ventricular
LVEDV: Left ventricular end-diastolic volume
LVEF: Left ventricular ejection fraction
MACE: Major adverse cardiovascular events
NT-proBNP: N-terminal prohormone B-type natriuretic peptide
PEDSR: Peak early diastolic strain rate
RVEF: Right ventricular ejection fraction
SGLT2: Sodium-glucose cotransporter-2
SSFP: Steady-state free precession
T2D: Type 2 diabetes.

## Additional Points

*Clinical Perspectives*. Competency in Medical Knowledge: in the absence of overt clinical coronary artery disease, uncontrolled hypertension, change in BMI, or HbA1c, T2D results in significant reductions in cardiac size and biventricular systolic function over time. There is a significant correlation of the change in cardiac function with baseline plasma hs-cTnT. Plasma hs-cTnT could, therefore, potentially serve as a screening tool to identify patients with T2D at higher heart failure risk. Translational Outlook 1: larger studies are needed to further delineate the relationship between plasma hs-cTnT with the risk of developing cardiac dysfunction in patients with T2D. As an easily obtainable plasma biomarker, hs-cTnT may be of use in the incremental risk stratification of patients with T2D into high- and low-risk subgroups and to translate prognostic projections into individualized management plans to address the excess heart failure risk in diabetes. Translational Outlook 2: the data from this study suggest that patients with T2D with higher LV mass and LV concentric remodeling might be at a higher risk of MACE. Aggressively targeting risk factors in patients with T2D with higher LV mass and/or LV concentric remodeling might lead to improved cardiovascular outcomes.
